# The Biophysical Properties of Basal Lamina Gels Depend on the Biochemical Composition of the Gel

**DOI:** 10.1371/journal.pone.0118090

**Published:** 2015-02-17

**Authors:** Fabienna Arends, Constantin Nowald, Kerstin Pflieger, Kathrin Boettcher, Stefan Zahler, Oliver Lieleg

**Affiliations:** 1 Institute of Medical Engineering IMETUM, Technische Universität München, Boltzmannstrasse 11, 85748, Garching, Germany; 2 Department of Mechanical Engineering, Technische Universität München, Boltzmannstrasse 15, 85748, Garching, Germany; 3 Department of Pharmacy—Center for Drug Research, Ludwig-Maximilians-Universität München, Butenandtstrasse 5–13, 81377, München, Germany; Center for Interdisciplinary Research in Biology (CIRB) is a novel Collège de France / CNRS / INSERM, FRANCE

## Abstract

The migration of cells within a three-dimensional extracellular matrix (ECM) depends sensitively on the biochemical and biophysical properties of the matrix. An example for a biological ECM is given by reconstituted basal lamina gels purified from the Engelbreth-Holm-Swarm sarcoma of mice. Here, we compare four different commercial variants of this ECM, which have all been purified according to the same protocol. Nevertheless, in those gels, we detect strong differences in the migration behavior of leukocyte cells as well as in the Brownian motion of nanoparticles. We show that these differences correlate with the mechanical properties and the microarchitecture of the gels which in turn arise from small variations in their biochemical composition.

## Introduction

In cell culture studies or tissue engineering applications, two different types of scaffolds are used to support cell proliferation, morphogenesis and differentiation: reconstituted matrices with purified biomacromolecules obtained from animal tissue or synthetic extracellular matrices (ECM). Both systems can be applied as surface coatings to promote cell adhesion, or they are used as a 3D environment for embedding cells to offer a more *in vivo*-like environment than a 2D system [1–3]. A critical advantage of an engineered scaffold is the opportunity to tune certain biophysical parameters, e.g. the mechanical properties or the permeability of the matrix and to investigate the influence of certain stimuli. On the other hand, ECM extracts from living cells may be better suited to approximate an *in vivo* environment in detail, especially if the biological macromolecules used to construct the scaffold are obtained from the same organism as the cells that are integrated into the matrix [4–6].

One of the simplest biopolymer model systems in use for constructing such a 3D scaffold is a reconstituted gel formed by collagen type I fibers. Macromolecules of the collagen family are key constituents found in various tissues such as the connective tissue (collagen type I), the basal lamina (collagen type IV) and cartilage (collagen type II) [7–9]. The major part of the collagen in animals consists of type I, II and III which forms long thin fibrils. Collagen type IV molecules on the other hand assemble into a two-dimensional reticulum [9, 10]. However, they are not the only macromolecular components in those tissues, so the predictive strength of results obtained from cell culture experiments in such simple collagen matrices is limited. Of course, the degree of variability and the ensuing range of biophysical properties of a scaffold comprising only one macromolecular component are much lower compared to a scaffold consisting of several components. A more complex multi-component ECM model system is given by basal lamina gels that are typically purified from murine tissue. The basal lamina is situated at the basolateral side of the endothelium and separates the endothelial cells from the connective tissue. This specialized extracellular matrix is composed of three main macromolecular constituents, i.e. laminin, collagen IV and the perlecan complex, the latter of which combines three heparin sulphate chains into a finger-like structure which is attached to the biopolymer network. In addition to those macromolecular components, the cross-linking protein entactin (nidogen) is found in those basal lamina gels where it connects the collagen network with the laminin macromolecules [11]. Thus, such a basal lamina gel offers a much higher complexity and biologically more relevant environment than simple collagen gels [8,12–14].

There are several commercially available variants of basal lamina gels, and those variants are all purified from Engelbreth-Holm-Swarm sarcoma tissue of mice. Nevertheless, it is common practice not to mix and match basal lamina gels from different vendors or batches for a set of experiments. This practice is mostly based on experience and the notion that, even though those basal lamina gels all originate from the same tissue type and the preparation procedure applied is based on the same protocol established by Kleinman et al.[15,16], variations in the gel properties might still occur and thus affect the experimental outcome. However, to our knowledge, those putative variations in the gel properties have not been systematically quantified yet. A detailed understanding of how variations in the biophysical properties of those gels are regulated on a molecular level and how the gel properties in turn affect the behavior of embedded cells is still missing. Such information will not only simplify the interpretation of cell culture experiments but also provide important insights for the design of synthetic hydrogel scaffolds with tailored biophysical properties.

Here, we use basal lamina gel preparations obtained from four different suppliers as a platform to investigate how the biophysical properties of these gels depend on their biochemical composition. We analyze the motility of differentiated HL-60 cells in those gels, quantify the Brownian motion of nanoparticles in the gel and the viscoelastic properties of the gels, and compare their biochemical constitution and microstructure. Although all gel variants tested have been purified from the same tumor tissue of mice, we detect strong differences in the gel properties ranging from decreased permeability to increased stiffness and mild cytotoxic behavior. Those differences in the gel properties can be traced back to small variations in the molecular composition of the gel preparations which lead to differences in the gel architecture.

## Materials and Methods

### Basal lamina gels

All basal lamina gels used in this study are growth factor reduced and have been purified from the Engelbreth-Holm-Swarm sarcoma of mice. We note that, besides the main macromolecular components described in the main text, those basal lamina gels are also likely to contain minor amounts of molecules that originate from other tissue types [17] which is due to the particular purification process. The gels were obtained from the following four suppliers: Sigma Aldrich (ECM1), BD Bioscience (ECM2), Trevigen (ECM3) and Life Technologies (Invitrogen) (ECM4). The protein concentration of the ECMs varied from c = 7.37 mg/mL (ECM1) to c = 15.65 mg/mL (ECM3) but was adjusted for the experiments to 3.5 mg/mL by dilution with Iscove’s Modified Dulbecco’s Medium (IMDM, PAA Laboratories GmbH, Pasching, Austria). Experimental results were checked with a second batch of gels from BD, Invitrogen and Trevigen. It was, however, not possible to obtain a second gel batch from Sigma-Aldrich. Thus, as an alternative, we used a non-growth factor reduced gel from Sigma to repeat all experiments except the cell migration studies, since the additional growth factors influence the outcome of this experiment and it would not be comparable to the results obtained before.

### Polystyrene particles

Fluorescent polystyrene latex particles (carboxyl-terminated or amine-terminated) with a diameter of 200 nm were obtained from Invitrogen. Polyethyleneglycol (PEG, M_w_ = 750 Da, Rapp Polymere, Tübingen, Germany) coating of fluorescent 200 nm carboxyl-terminated latex beads was performed using a carbodiimide-coupling protocol [18]. Successful PEGylation was verified by determining the zeta-potential of the particles using dynamic light scattering implemented in a Zetasizer ZS (Malvern Instruments, Herrenberg, Germany). When suspended in 20 mM HEPES (Carl Roth, Karlsruhe, Germany) buffer at pH 7, we measured a zeta-potential of ζ = -38 mV for the carboxylated particles before PEGylation and ζ = -20 mV after PEGylation.

### Particle diffusion experiments

For particle diffusion experiments, the ECM gels were thawed on ice and afterwards diluted with IMDM to a final protein concentration of 3.5 mg/mL. To induce gelation, all samples were incubated at their final protein concentrations in presence of the respective test particles at 37°C for 30 min. Particle trajectories were obtained and analyzed as described before [19]. In brief, movies of particles were acquired with a digital camera (Orca Flash 4.0 C11440, Hamamatsu, Japan) using the software Hokawo provided by Hamamatsu on an Axiovert 200 (Zeiss, Oberkochen, Germany) microscope with a 32 x objective (Zeiss, Oberkochen, Germany). The particle position was then determined for each frame by fitting a Gaussian to the x- and y-section of the intensity profile of each particle. Then, the mean-square displacement (MSD) was determined from the trajectory $\vec{r}(t)$ of a particle, as follows:

$$MSD(\tau )=\frac{1}{N}{\sum_{i=1}^{N}\left[\vec{r}\left(i\Delta t+\tau \right)-\vec{r}(i\Delta t)\right]}^{2}$$

Assuming normal diffusion, the mean-square displacement is related to the diffusion coefficient via *MSD*(*τ*) = *2nDτ*, where *n* = 2 applies for the quasi-two-dimensional trajectories $\vec{r}(t)=\left(x(t),y(t)\right)$ analyzed here. All particles with an apparent diffusion coefficient larger than *D*_cut_ = 1 μm^2^/s, which is half the diffusion coefficient of a 200 nm-sized particle in water, were classified as “diffusing”. In every sample, particles from at least three different fields of view were analyzed, and every experiment was repeated three times. That way, a total of at least 1000 particles were analyzed for each particle species.

### Cell migration experiments

For cell migration experiments, the human promyelocytic leukemia cell line HL-60 (CCL-240, ATCC, Wesel, Germany) was used. This cell line was developed as a simple model system to study neutrophil cell migration without the need to derive cells from primary tissue [20]. HL-60 cells have several advantages over primary neutrophils, which include a significantly longer life span and a higher reproducibility in their behavior. HL-60 cells can be maintained in culture, and can be terminally differentiated into migration-competent neutrophil-like cells (dHL-60) using dimethylsulfoxide (DMSO, Carl Roth, Karlsruhe, Germany). In brief, HL-60 cells were cultivated in IMDM supplemented with 15% heat-inactivated fetal bovine serum (FBS, PAA Laboratories GmbH, Pasching, Austria) at 37°C and 5% CO_2_. Cells were grown in suspension until they reached a density between 1-2x10^6^ cells/mL before they were passaged. To differentiate cells, 1.3% (v/v) DMSO was added to 2x10^5^ cells/mL suspended in fresh IMDM+FBS. Upon differentiation, cells underwent clear morphological changes which were detected under a light microscope. Because cells are most active 4-5 days post-differentiation they were used after 4 days for cell migration experiments. An amount of 1x10^4^ dHL-60 cells were suspended in ice-cold ECM samples as obtained from the four different suppliers and diluted with IMDM supplemented with N-formyl-methionine-leucine-phenylalanine (fMLP, Sigma-Aldrich, Schnelldorf, Germany) to a final concentration of 3.5 mg/mL ECM and 50 nM fMLP. The uniformly distributed chemoattractant fMLP triggered spatially homogeneous cell migration. A volume of 50 μL of each ECM/cell mixture was then transferred into one lane of a μ-Slide VI 0.4 (ibidi, Planegg/Martinsried, Germany) and incubated for 30 min at 37°C and 5% CO_2_ in a cell incubator to allow for gel formation. After gelation, 50 μL of IMDM supplemented with 50 nM fMLP were added on top of the gel both at the inlet and outlet of the lane to allow for continuous nutrient supply and to minimize drying effects. Image acquisition for the migration experiments was performed on an Axiovert 200M microscope (Zeiss, Oberkochen, Germany) using a 10 x objective (Zeiss, Oberkochen, Germany). An incubation chamber mounted onto the microscope was used to control temperature and CO_2_ concentration. Movies of migrating cells were recorded with an AxioCam digital camera (Zeiss, Oberkochen, Germany) using the software AxioVision V.4.8.20 (Zeiss, Oberkochen, Germany). To avoid artefacts arising from gel swelling, an initial adjustment time of 4 hours was provided. Then, phase contrast images were acquired every minute for 2 hours at different gel locations. The position of the cells in the gel matrices was determined with ImageJ 1.47d for every frame of the migration video, and the migration velocity was then calculated by multiplying the average migrated distance per frame with the frame rate. The Euclidean distance (*ED*) between the cell position at the start and the end of the evaluation was calculated with Chemotaxis and Migration Tool V2.0 (ibidi, Planegg/Martinsried, Germany) and averaged over all cells in the respective gel.

### Rheology

The quantification of the viscoelastic properties as well as the gelation kinetics of the different ECM gels was performed on a stress-controlled macrorheometer (MCR 302, Anton Paar, Graz, Austria) with a 25 mm plate-plate geometry at a plate separation of 200 μm using a torque of 0.5 μNm and a frequency of 1 Hz ensuring linear response. The rheometer plate was cooled to 5°C before 150 μL of the samples were added, and gelation was induced by a sudden temperature change to 37°C. A thin layer of polydimethylsiloxane oil (ABCR, Karlsruhe, Germany) was applied to the outer rim of the sample to avoid drying artefacts. An applied oscillatory stress σ = σ_0_ sin(*⍵*t) with a frequency *⍵* resulted in an oscillatory strain with the same frequency, *γ* = *γ*_0_ sin(*⍵*t + *δ*), where δ denotes the phase shift between stress σ and strain γ. With those parameters, the storage modulus *G‘* = σ_0_ / *γ*_0_ cos(*δ*) can be calculated which is a measure for the elastic properties of the gel, and the loss modulus *G”* = σ_0_ / *γ*_0_ sin(*δ*) which is a measure for the viscous properties of the gel.

### Western blots

For detection of proteins in western blot the following primary antibodies were used: the mouse monoclonal anti-fibronectin C6F10 (1:200 dilution), the rat monoclonal anti-nidogen ELM1 (1:500 dilution) (sc-73611, sc-33706; Santa Cruz, Heidelberg, Germany), the rabbit polyclonal anti-collagen type IV (1:200 dilution) (AB756P; Millipore, Darmstadt, Germany) and the rabbit polyclonal anti-laminin (1:500 dilution) (L9393; Sigma-Aldrich, St.Louis, USA). The following secondary antibodies were used: anti-mouse IgG, HRP-linked (1:2000 dilution) (7076; Cell Signaling Technologies, Cambridge, UK), anti-rabbit IgG (H+L), HRP-linked (1:2000 dilution) (111–035–144; Dianova, Hamburg, Germany), anti-rat IgG (H+L), HRP-linked (1:2000 dilution) (6180–05; SouthernBiotech, Birmingham, USA) and anti-rabbit IgG (H+L), IRDye 800 (1:5000 dilution) (611–132–122; Rockland, Gilbertsville, USA). The ECM gels were thawed on ice and heated in Laemmli buffer at 95°C for 5 min. Equal amounts of protein were loaded on polyacrylamide gels. The proteins were separated by SDS-PAGE and transferred to nitrocellulose membranes using tank blotting. For the detection of protein levels, the ECL detection system (Amersham Pharmacia Biotech, Uppsala, Sweden) or Odyssey Infrared system version 2.1 (LI-COR Biosciences, Lincoln, USA) was used.

### Confocal microscopy

The following antibodies were used for imaging applications: the goat polyclonal anti-collagen type IV (1:100 dilution) (sc-167526; Santa Cruz, Heidelberg, Germany) and the Alexa Fluor 680 donkey anti-goat IgG (H+L) (1:200 dilution) (A-21084; Life Technologies, Carlsbad, USA). For determining the gel microarchitecture with confocal microscopy, the ECM gels were thawed on ice and afterwards diluted with DMEM (Life Technologies, Carlsbad, USA) to a final protein concentration of 3.5 mg/mL. The gels were stained in μ-Slide Chemotaxis^3D^ (ibidi, Planegg/Martinsried, Germany). 6 μL of gel were injected into the observation channel of the slide and incubated for 30 minutes at 37°C, 5% CO_2_. Afterwards the gels were fixed with 2% glutaraldehyde for 40 minutes and blocked with 1% bovine serum albumin (BSA) for 24 hours at 4°C. Subsequently, gels were incubated with primary antibodies, diluted 1:100 with 1% BSA for 72 hours at 4°C. Samples were washed three times with PBS (20 minutes) before incubation with secondary antibodies, diluted 1:200 with 1% BSA for 48 hours at 4°C. Images were obtained using a SP8 SMD confocal microscope (Leica, Wetzlar, Germany) and a 63 x HC PL APO 1.2 NA water objective (Leica, Wetzlar, Germany). The thickness of the optical slices was 0.9 μm.

### Scanning electron microscopy

For scanning electron microscopy (SEM, JEOL-JSM-6060LV, Jeol, Eching, Germany) images, the gels were thawed on ice and diluted afterwards with IMDM to a final protein concentration of 3.5 mg/mL. A volume of 30 μL was pipetted onto a sample holder and incubated for 30 min at 37°C to induce gelation. The samples were fixed in 2.5% glutaraldehyde (in 50 mM HEPES, pH 7.4) for one hour and washed with ddH_2_O for another hour. For dehydration, the samples were incubated in an increasing ethanol series of 50%, 70%, 80% and 99.8% ethanol for 30 min, each. Then, the samples were critical point dried, sputtered with a conductive gold film (40 mA, 40 s) and imaged at 5 kV.

### Life-dead assay

For the life-dead assay tissue-culture treated 96-well plates were loaded with 50 μL of the different ECM gels at a final concentration of 3.5 mg/mL containing 50 nM fMLP and 1×10^4^ dHL-60 cells as described above (see cell migration section). After gel formation for 30 min at 37°C and 5% CO_2_ in a cell incubator 100 μL fresh IMDM containing 15% FBS and 50 nM fMLP were added on top to prevent drying effects. After additional 24 h of incubation in a cell incubator a live-dead staining was performed. For this 150 μL of a 4 μM calcein-AM/ethidium homodimer-1 mix (Life Technologies, Darmstadt, Germany) in IMDM was added. Calcein-AM is green fluorescent in its activated conformation in the cytoplasm of vital cells activated by intracellular esterases. Ethidium homodimer-1 binds to DNA in the cell nucleus of dead cells possessing a damaged cell membrane. Accordingly, green appearing vital cells can easily be distinguished from dead red cells and counted by means of a fluorescent microscope. Image acquisition was performed after 1 h of incubation on an Axiovert 200M (Zeiss, Oberkochen, Germany) microscope using a 10 x objective (Zeiss, Oberkochen, Germany). Images were analyzes using ImageJ 1.47d.

### Mass spectroscopy

The ECM gels were thawed on ice and heated in Laemmli buffer supplemented with 250 mM DTT (Sigma-Aldrich, Schnelldorf, Germany) at 95°C for 5 min. An amount of 30 μg of protein were loaded on a 4 – 20% gradient polyacrylamide gel (Bio Rad, München, Germany) respectively. The proteins were separated by SDS-PAGE and stained with a coomassie brilliant blue R-250 staining solution (Bio Rad, München, Germany). The band of interest was cut out and sent to the chemistry department (TUM, Garching, Germany) for trypsin digestion and subsequent MALDI TOF/TOF fingerprint mass spectroscopy.

## Results and Discussion

### Cell migration studies

**Fig 1:**
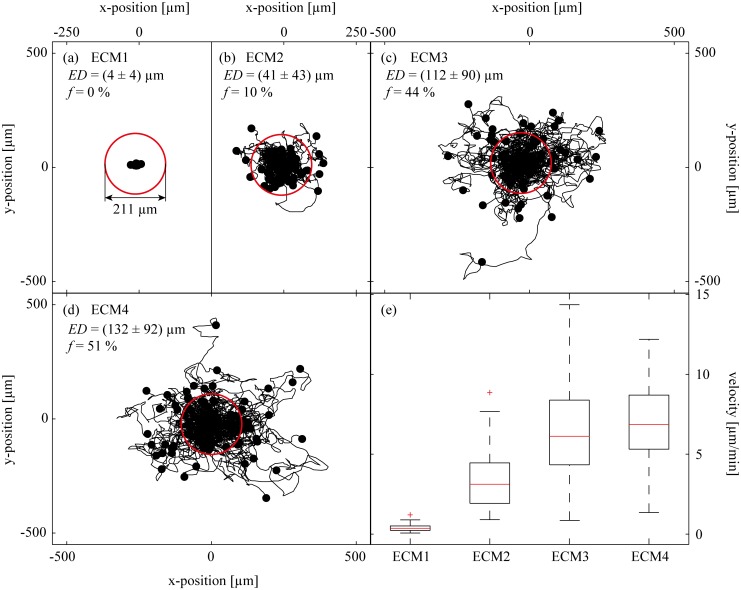
Migration trajectories of dHL-60 cells tracked from hours 4–6 after the cells are embedded into the four different basal lamina matrices. The starting point of all trajectories is shifted to the origin for clarity, and the end point is marked by a dot. The average start-to-end distance (Euclidean distance) travelled by the cells and the respective standard deviation is denoted by *ED*. The fraction of cells with an *ED* greater than *ED* = 105.5 μm (red circle) is denoted by *f*. (e) Comparison of the migration velocity of dHL60 cells in different ECM gels. The red line denotes the median of the velocity distribution, the box includes 25% of the observed velocities above and below this median, respectively. The remaining 25% of slower as well as the 25% of faster cells are indicated by the dashed lines. Outliers are denoted by a red cross.

Reconstituted extracellular matrices such as basal lamina gels are mostly used for embedding cells and analyzing their migration behavior as a function of distinct knock-out mutations or external (bio)chemical stimuli. There, the unperturbed migration activity of the cells in the gel matrix is typically used as a reference. Thus, in a first step, we conduct 3D cell migration experiments with dHL-60 cells using the four different ECM systems. Together with the cells, the chemoattractant fMLP is added to the gels before gelation is induced. Fig. 1 shows the trajectories of migrating dHL-60 cells as obtained 4 hours after gelation of the ECMs. This delayed observation time is chosen to exclude an influence of gel swelling (as it occurs during the first hours of the experiment) on the cell migration data. Each trajectory shown denotes the time-dependent cell position for a time course of 2 hours. The start point of all trajectories is shifted to the origin for clarity, and the end point is marked by a dot. As depicted in Fig. 1, the cells are able to efficiently migrate in ECM2, ECM3 and ECM4. Due to the uniform distribution of the chemoattractant, we observe random cell migration without any spatially oriented preference. The area covered by the trajectories varies depending on the gel used and is a measure for the migration activity. This area is similar for ECM3 and ECM4 whereas it is significantly smaller for ECM2. For ECM1, we are not able to detect any migratory activity, and the area covered by the trajectory ensemble is on the order of our tracking error. Another measure for the migratory activity is the Euclidean distance (*ED*) which is only *ED* = (4 ± 4) μm for cells embedded in ECM1 and *ED* = (41 ± 43) μm for ECM2. Again, ECM3 and ECM4 return very similar results with *ED* = (112 ± 90) μm for ECM3 and *ED* = (132 ± 92) μm for ECM4. An analysis of this data shows that only a fraction of *f* = 10% of the cells embedded in the ECM2 have an Euclidean distance greater than 105.5 μm (depicted by a red circle in Fig. 1) whereas in the gels of ECM3 and ECM4 we find *f* = 44% and *f* = 51%. In ECM1 no cell has an ED greater than 105.5 μm, therefore *f* = 0%. To further quantify those differences in the migratory behavior of the dHL-60 cells, the cell migration speed is calculated from the individual trajectories. When this velocity data is pooled for each cell population and compared in a box plot (Fig. 1e), the following differences between the distinct gel environments are observed: First, the median velocity of cells within ECM3 and ECM4 is higher than in ECM2, and the migration speeds determined for cells in ECM1 is close to zero. These findings agree well with our analysis of the total area explored by the migrating cell populations and the *ED*. Second, the distribution of migration speeds is quite broad both in ECM3 and ECM4, but more homogeneous in ECM2. Together, this data suggests that ECM3 and ECM4 offer the most efficient environment for dHL-60 migration whereas ECM2 seems to slow down the cellular migration activity and ECM1 completely suppresses dHL-60 cell migration. Apart from the suppressed migration we observe more dead cells in the ECM1 than in the other ECMs. To quantify this observation, a live/dead assay is performed. We determine a fraction of dead cells in the range of 5–10% for ECM2, ECM3 and ECM4, but at least twice as much, i.e. about 20% dead cells, are observed for ECM1 (see S1 Fig.). Whereas this finding already demonstrates that there are significant differences between the four gel variants, it cannot explain the differences in permeability of the gels towards migrating cells as only living cells are included in our analysis.

### Particle diffusion and gel microarchitecture

**Fig 2:**
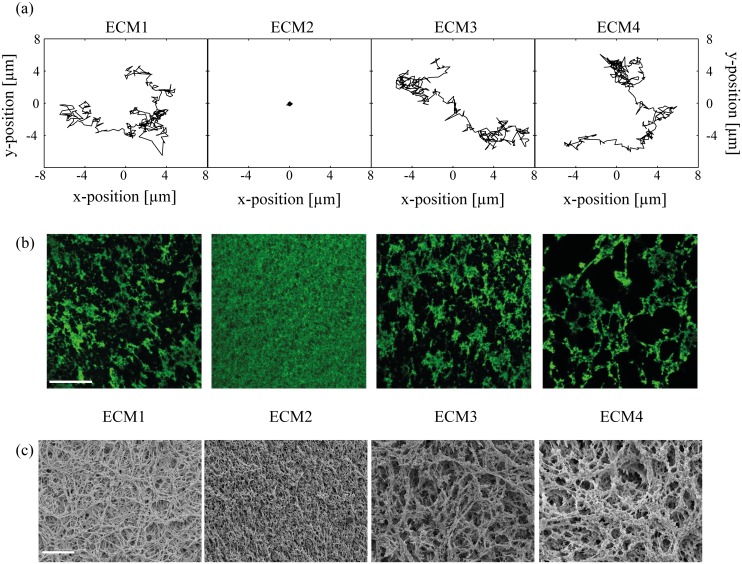
Micromorphology of the four gels determined by three different methods. (a) Exemplary trajectories of PEGylated particles with a diameter of 200 nm in the four different gels. Trajectories are shifted for clarity. (b) Micromorphology of the four different basal lamina gel variants as determined by confocal fluorescence microscopy. Representative staining of the matrix component collagen IV. The scale bar in the upper left image denotes 50 μm and applies to all images. (c) Micromorphology of the whole network of the four gel variants imaged by SEM. The scale bar corresponds to 25 μm and applies to all images.

As a next step, we further quantify the microscopic permeability properties of the different gels. Therefore, we choose fluorescent 200 nm-sized polystyrene beads as tracer particles, embed them into the gels and investigate their Brownian motion. Two important gel properties can be derived from such diffusion experiments with nanoparticles: First, information about the gel microstructure can be obtained when passivated nanoparticles are used that do not bind to the gel constituents. Second, the selective properties of the gel matrix can be mapped when particles with identical size but different surface modifications are compared [21]. Accordingly, we here compare the diffusion behavior of polystyrene particles carrying either COOH, NH_2_ or polyethylenglycol (= PEG) groups on their surface. In a previous study with ECM1, we have observed that only particles with neutral or weakly charged surfaces are able to diffuse through those gels—provided that their size is smaller than the mesh size, i.e. the average spacing between neighboring macromolecule strands of the gel. However, the diffusion of strongly charged particles can be completely suppressed even for small particles. This behavior was attributed to binding events of the particles to the gel constituents [19,22]. Indeed, we observe similar charge dependent diffusion behavior here. We find that particles with COOH and NH_2_ surface groups are immobilized in all gels demonstrating that the ECMs exhibit similar selective permeability properties towards charged nanoparticles. In contrast, the experiments with PEGylated particles show both diffusing and immobilized particles and exemplary trajectories are shown in Fig. 2a. The fraction of diffusing particles was (80 ± 1) % in ECM1, (77 ± 3) % in ECM3 and (73 ± 4) % in ECM4 (see also S1 Table). In contrast, all of those PEGylated particles are completely immobilized in ECM2. As particle PEGylation is expected to shield nanoparticles from adhesive interactions established by both electrostatic and hydrophobic forces [23–25], this suggests that the microarchitecture of ECM2 might be significantly different from that of the other gel variants, and that the Brownian motion of the PEGylated particles is obstructed by geometric hindrance effects imposed by a small mesh size.

If the average mesh size of ECM2 is indeed significantly smaller than in the other three gel variants, we might be able to directly detect differences in the gel architecture using fluorescence microscopy. To visualize the micromorphology of the four gels, collagen is stained with antibodies and images of the gel structure are obtained with a confocal microscope. The results shown in Fig. 2b confirm our previous notion based on the particle diffusion experiments: although all gels are diluted to the same final protein concentration of 3.5 mg/mL, ECM2 shows a much more homogeneous microstructure and a smaller mesh size than the other ECMs (for a higher magnification see S2 Fig. and for a second batch see S3 Fig.). However, the microstructure of ECM1, ECM3 and ECM4 appears to be very similar to each other with pores that seem to be at least tenfold larger than those found in ECM2.

To consolidate our findings obtained by fluorescence microscopy, we next analyze the microarchitecture of the gels by using scanning electron microscopy (SEM). In contrast to fluorescence microscopy, the whole network instead of only one component of the network is visualized. Fig. 2c and S2 Fig. show the SEM images obtained for the four gels. Again, ECM1, ECM3 and ECM4 have a similar microarchitecture whereas ECM2 shows a smaller pore size. In general, the mesh size of a gel can be decreased by increasing the concentration of protein. However, in our experiments the total protein amount is fixed at 3.5 mg/mL for all four gels, accordingly a smaller mesh size can only be achieved by thinner strands. Indeed, the fibers setting up the local meshwork in ECM2 are thinner than in the other gels and seem to contain less molecules per strand.

### Viscoelastic properties of the gels

As the four gels seem to differ significantly in terms of their mesh size, one would also expect differences in their macroscopic viscoelastic properties. This expectation is based on our experience with other biological hydrogels where the macromechanical properties of the gels depend on a range of factors. Those parameters include the concentration and micromechanical properties of the macromolecules, and the microarchitecture of the gel. The latter is described by the type of spatial configuration in the gel—be it a homogeneous or heterogeneous distribution of elements—and the mesh size [26,27]. We here assess the influence of the mesh size variations observed between ECM2 and the three other variants by quantifying the viscoelastic properties of the gels with a macrorheometer. Such a rheometer exerts shear forces on a given sample and measures the resulting sample deformation. From these two quantities, the storage modulus *G*’ as well as the loss modulus *G*” is calculated, where the former serves as a measure for the elastic properties of the material and the second gives a measure for the viscous properties of the sample.

**Fig 3:**
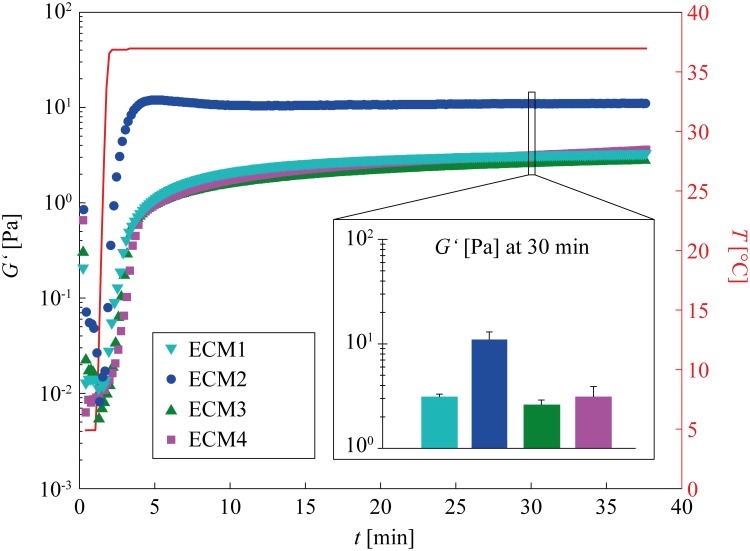
Gelation kinetics of the four different gels measured with a macrorheometer. The temperature is increased from 5°C to 37°C after one minute to induce gelation. The curves shown represent averages of three independent measurements. The inset shows the storage moduli *G’* of the four gels at 30 min. The error bars denote the error of the mean.

The ECM gels used here are typically stored as a liquid at temperatures of 5°C or below and are known to form a viscoelastic gel at physiological temperatures. Thus, we first investigate the gelation kinetics of the gels. According to the manufacturers’ information, all ECM variants should gel within the first few minutes when the samples are heated to 37°C. Again, all basal lamina variants are adjusted to the same total protein concentration of 3.5 mg/mL for experimental comparison. We observe efficient gel formation for all four gel variants, and no significant differences in the gelation kinetics are detected (Fig. 3).

Second, we compare the final elasticity the gels reach after complete gelation. We find that ECM1, ECM3 and ECM4 reach nearly identical final elasticities of *G*’ ~ 3 Pa, whereas ECM2 shows a fourfold higher stiffness (inset of Fig. 3 and S4, S5 Figs.). This agrees with our notion that differences in the gel mesh size should manifest themselves in the viscoelastic properties of the gel.

### Molecular composition of the gels

In our comparison of the four different basal lamina gel variants, we have observed strong differences in the migration activity and viability of embedded dHL-60 cells, the Brownian motion of nanoparticles and the macromechanical properties. Those findings are surprising considering that all gels are adjusted to the same total protein concentration and identical experimental conditions are chosen for each set of experiments: all cell migration experiments are conducted with the same cell population on the same microscope within an incubation chamber where the temperature as well as CO_2_ concentration are controlled. Indeed we find that the gel stiffness, cell migration speed and particle diffusion correlate to a certain degree, and that this can be largely attributed to differences in the gel microarchitecture: small pore sizes cause higher gel stiffness and increased hindrance towards migrating cells and diffusing particles (ECM2), while larger pores facilitate migration and diffusion (ECM3, ECM4). The only discrepancy in this correlation occurs for ECM1: here, on the one hand, mesh size, particle diffusion and stiffness are comparable with those of ECM3 and ECM4; on the other hand, the cell migration efficiency differs dramatically as compared to these gels.

**Fig 4:**
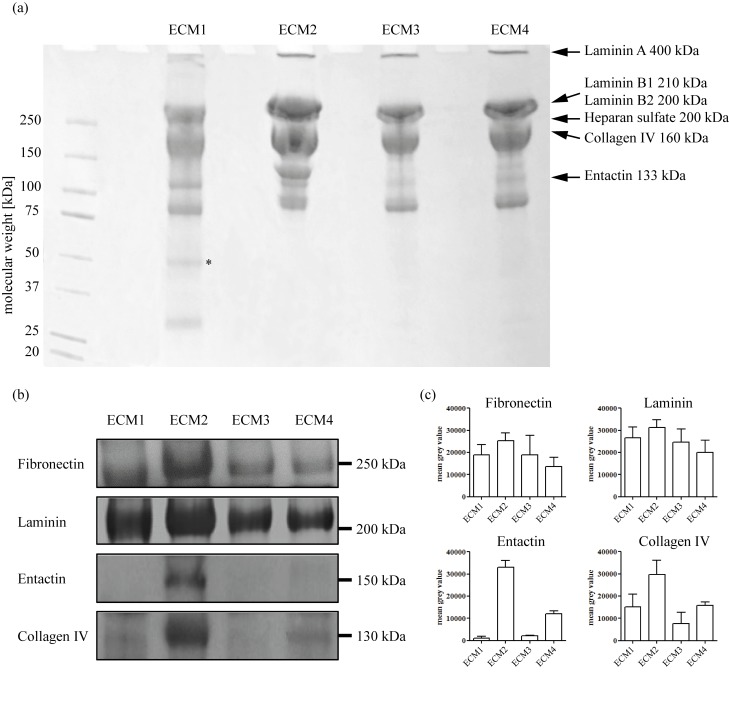
Content of selected ECM proteins in the four different ECM gel variants. (a) A coomassie staining of the four gel variants shows extra bands in ECM1 at low molecular weight. The star denotes the band which is further investigated by mass spectroscopy (see S2 Table for details). (b) The content of fibronectin, laminin, entactin and collagen type IV in the four different ECM gels is analyzed by western blot (for uncropped blots see S7 Fig.). (c) Densiometric analysis of fibronectin, laminin, entactin and collagen IV signals. The error bars denote the standard deviations as obtained from four independent gel runs.

In order to address this puzzling observation we examine the composition of the gels by electrophoresis. Whereas the overall pattern of protein bands in the coomassie staining is similar in all four gels (Fig. 4a and S6 Fig.), we detect some additional bands in ECM1, which are further analyzed by a proteomics approach. From mass spectroscopy analysis, the laminin subunits alpha1 and beta1 are identified in the additional band from ECM1 together with some proteins unrelated to the extracellular matrix (see S2 Table). These laminin subunit proteins have a molecular weight around 300 kDa and 200 kDa, respectively [28], but are detected at a size of approximately 50 kDa. This suggests that a proteolytic breakdown has occurred during the purification process. Already in the early times of laminin research, cellular activities of proteolytic laminin fragments with exposed cryptic motifs have been described [29]. This has been meanwhile corroborated by many publications [30,31]. However, a clear and systematic analysis of biological effects of the different fragments is still missing. We can only speculate that due to the manufacturing procedure proteolytic cleavage of laminin might have occurred in ECM1 and that the resulting fragments perhaps are responsible for the suppressed cell migration (e.g. via increased substrate adhesion).

Still, the question remains why ECM2 would be stiffer than the other gels. The stiffness of a biopolymer-based hydrogel is governed by several parameters: First, the concentration of biopolymers determines the elasticity, the more polymers the higher this value. However, the concentration of biological macromolecules with high molecular weight is comparable for all gels when the total protein concentration is adjusted—which rules out this simple explanation. Second, the amount of cross-linking molecules determines the stiffness of the gel: the gel stiffness is increased with increasing concentrations of cross-linking molecules. Therefore, the higher stiffness of ECM2 could be due to a higher concentration of entactin, which acts as cross-linker between laminin and collagen IV in the basal lamina. The coomassie staining already hints towards quantitative differences in the gel composition which are further analyzed by western blot. The comparison of four main matrix constituents (fibronectin, laminin, entactin, and collagen IV) shows that in tendency all of them are more concentrated in ECM2 (Fig.4b, Fig. 4c, S7 Fig. and S8 Fig.). The most dramatic difference is detected for entactin, which is nearly absent in ECM1, ECM3 and ECM4. This finding supports our previous notion that indeed the entactin concentration is responsible for the observed differences in macromechanical properties and also in the microarchitecture of the gels.

## Conclusion

Here, we have investigated four commercially purified reconstituted variants of the basal lamina which were obtained from the same murine tissue following similar purification protocols. We detected variations in the concentration of the cross-linking protein entactin which entailed altered macromechanical properties as well as differences in the microarchitecture of the gels. This led to differences in the migration activity of dHL-60 cells and the Brownian motion of nanoparticles. Our findings demonstrate how sensitively the biophysical properties of a multi-component scaffold can depend on the concentration of a single component. In fact, entactin—compared to the content of the other macromolecular components of the basal lamina, i.e. laminin, collagen IV and heparan sulfate—is often considered to be a minor player only. Our results also suggest that proteolytic break-down of laminin observed in one of the gel variants led to the presence of protein fragments that severely hampered leukocyte migration activity—a mechanism that might also play an important role *in vivo*.

## Supporting Information

S1 Fig Life dead assay for dHL-60 cells embedded in the four basal lamina variants. Dead cells are obtained in all gels, but the amount differs drastically between ECM1 (about 20% dead cells) and the other three variants (about 5-10% of dead cells).
(DOCX)

S2 Fig Micromorphology of the four gel variants. (a) Magnification of the images obtained by fluorescence microscopy. The bar corresponds to 10 μm and applies to all images in the first row. (b) Images obtained with SEM at magnifications of 500x and 5000x. The scale bar in the upper row corresponds to 50 μm and in the lower row to 5 μm.
(DOCX)

S3 Fig Micromorphology of the second batch of the ECM variants as determined by confocal fluorescence microscopy. Representative staining of the matrix component collagen IV. The scale bar in the upper left image denotes 50 μm and applies to all images.
(DOCX)

S4 Fig Viscoelastic frequency spectrum obtained for the basal lamina variants. The storage moduli G’ (full circles) dominate over the loss moduli G” (empty circles) for all gel variants. Moreover, the storage moduli of all gels are nearly constant over two decades of frequency. This allows us to define a plateau modulus *G*_0_ for each of the gels: *G*_0, ECM1_ = 3 Pa, *G*_0, ECM2_ = 11 Pa, *G*_0, ECM3_ = 3 Pa, *G*_0, ECM4_ = 4 Pa. The measurement was performed on a stress-controlled macrorheometer (MCR 302, Anton Paar, Graz, Austria) with a 25 mm plate-plate geometry at a plate separation of 200 μm after 30 min gelation time at 37°C.
(DOCX)

S5 Fig Rheological results for the second batch of the gels. Again, ECM2 shows a significant higher *G’* than the other three gel variants. The error bar denotes the error of the mean.
(DOCX)

S6 Fig SDS-PAGE for second batch. There was no second batch of growth factor reduced ECM1 available therefore we used non-growth factor reduced (ngfr) ECM1 as a control. In ECM1 there is an additional band at 50 kDa which was not detected in the other ECMs.
(DOCX)

S7 Fig Uncropped blots for detection of the ECM proteins entactin, laminin, collagen IV and fibronectin. The entactin blot shows non-specific bands at 130 kDa, 110 kDa and 100 kDa, which were identified by Paulsson et al (*Purification and structural characterization of intact and fragmented nidogen obtained from a tumor basement membrane*, Eur. J. Biochem. 156, 467–478 (1986)), as entactin fragments obtained under conditions with less stringent control of endogenous proteolysis. The unspecific bands occurring in the laminin blot might show laminin B1 and B2 (>200 kDa) as well as shorter proteolytic laminin fragments (130 and 72 kDa). Short proteolytic fragments might also occur in the collagen IV blot (72 kDa). This blot additionally shows non-specific bands in the region between 300 kDa and 180 kDa, which might be explained by cross-reactivity of the collagen IV-antibody with laminin and fibronectin. The non-specific bands in the fibronectin blot might be also due to short proteolytic fragments.
(DOCX)

S8 Fig The content of fibronectin, laminin, entactin and collagen type IV in the second batch of the four different ECM gels is analyzed by western blot. Densiometric analysis of fibronectin, laminin, entactin and collagen IV signals. The error bars denote the standard deviations as obtained from three independent gel runs. ECM2 shows again the significantly highest amount of entactin.
(DOCX)

S1 Table Analysis of particle tracking experiments for a second batch of the gels. Again, amine-terminated as well as carboxyl-terminated particles are immobile in all ECMs. ECM1, ECM3 and ECM4 show a similar fraction of diffusing PEGylated particles whereas in ECM2 only immobile particles are detected.
(DOCX)

S2 Table Results from mass spectroscopy analysis of the additional band of ECM1. Database: NCBInr 20140323, Taxonomy: Mus musculus, Type of search: Peptide mass fingerprint, Enzyme: Trypsin
(DOCX)
