# Supplementary material for: The Biophysical Properties of Basal Lamina Gels Depend on the Biochemical Composition of the Gel
Source: PLoS One. 2015 Feb 17;10(2):e0118090. doi: 10.1371/journal.pone.0118090 (PMC4331274; doi:10.1371/journal.pone.0118090)
Supplement: S1 Fig — Dead cells are obtained in all gels, but the amount differs drastically between ECM1 (about 20% dead cells) and the other three variants (about 5-10% of dead cells). (DOCX) [file pone.0118090.s001.docx]

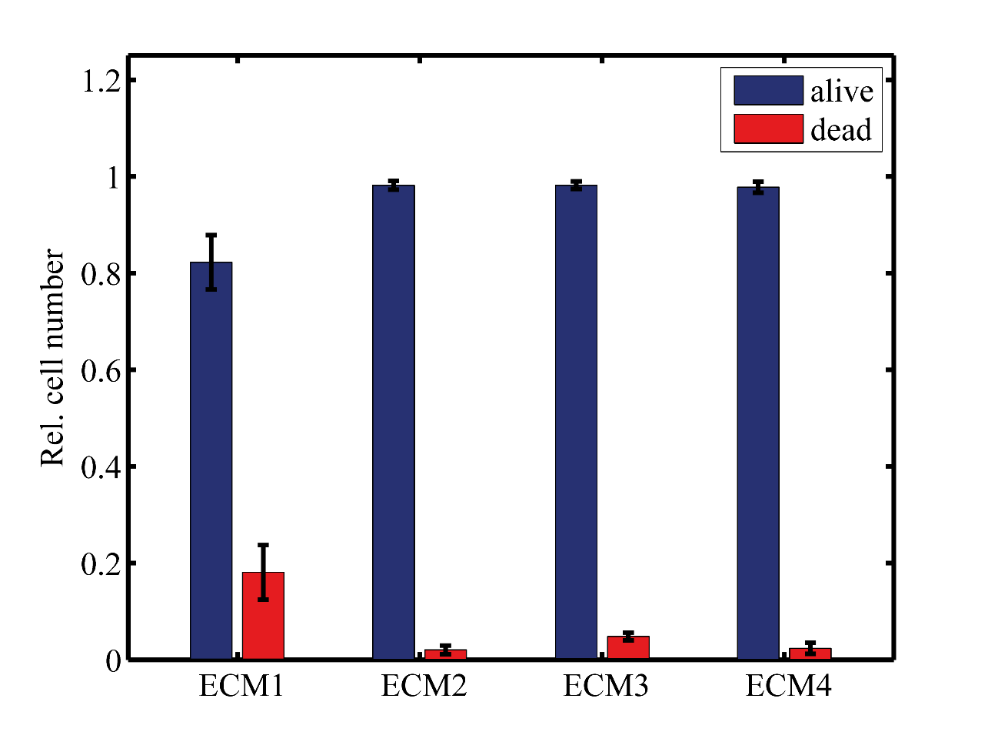


**Figure S1.** Life dead assay for dHL-60 cells embedded in the four basal lamina variants. Dead cells are obtained in all gels, but the amount differs drastically between ECM1 (about 20 % dead cells) and the other three variants (about 5‑10 % of dead cells).
