# Supplementary material for: The Biophysical Properties of Basal Lamina Gels Depend on the Biochemical Composition of the Gel
Source: PLoS One. 2015 Feb 17;10(2):e0118090. doi: 10.1371/journal.pone.0118090 (PMC4331274; doi:10.1371/journal.pone.0118090)
Supplement: S2 Fig — (a) Magnification of the images obtained by fluorescence microscopy. The bar corresponds to 10 μm and applies to all images in the first row. (b) Images obtained with SEM at magnifications of 500x and 5000x. The scale bar in the upper row corresponds to 50 μm and in the lower row to 5 μm. (DOCX) [file pone.0118090.s002.docx]

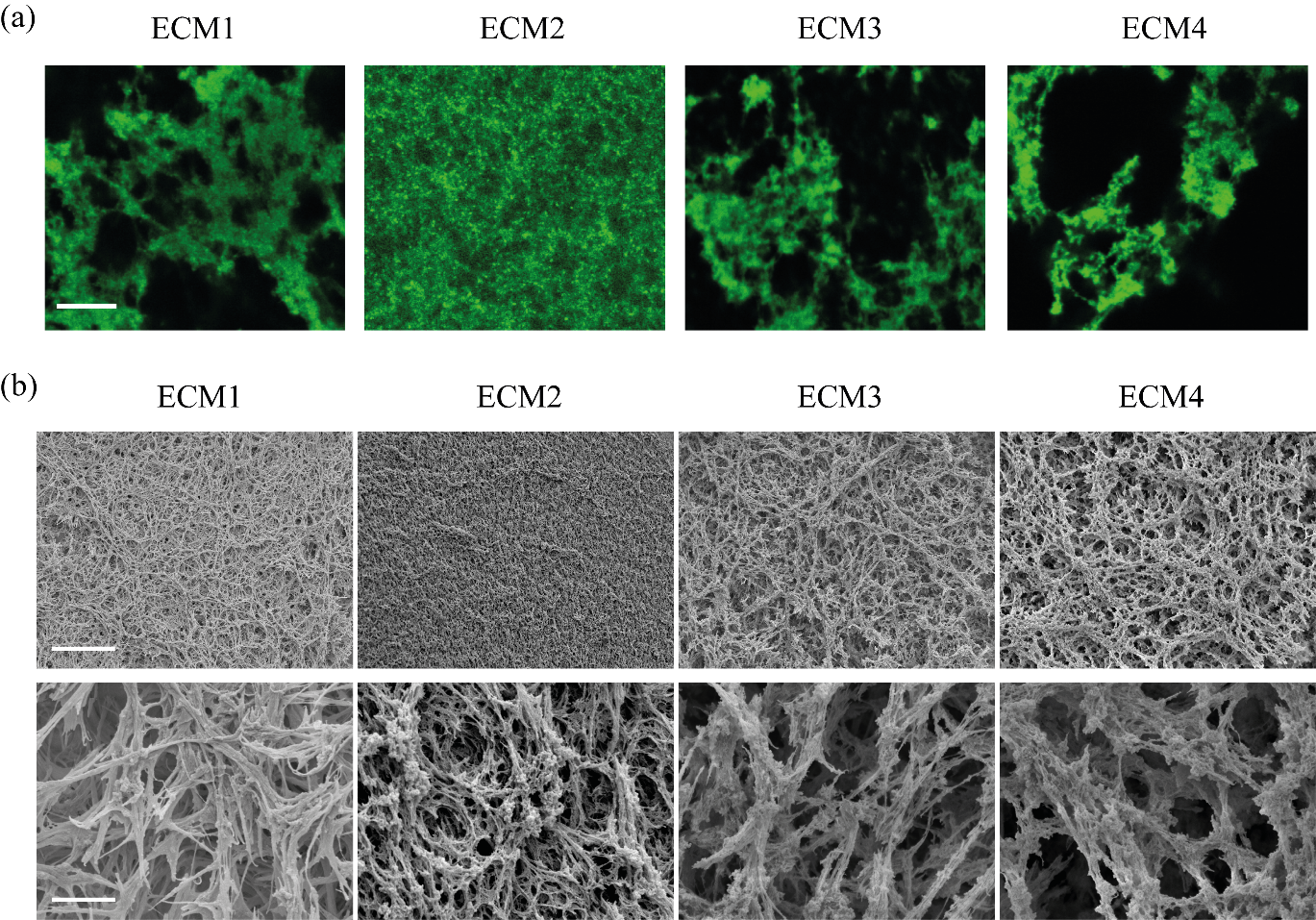


**Figure S2.** Micromorphology of the four gel variants. (a) Magnification of the images obtained by fluorescence microscopy. The bar corresponds to 10 µm and applies to all images in the first row. (b) Images obtained with SEM at magnifications of 500x and 5000x. The scale bar in the upper row corresponds to 50 µm and in the lower row to 5 µm.
