# Supplementary material for: The Biophysical Properties of Basal Lamina Gels Depend on the Biochemical Composition of the Gel
Source: PLoS One. 2015 Feb 17;10(2):e0118090. doi: 10.1371/journal.pone.0118090 (PMC4331274; doi:10.1371/journal.pone.0118090)
Supplement: S3 Fig — Representative staining of the matrix component collagen IV. The scale bar in the upper left image denotes 50 μm and applies to all images. (DOCX) [file pone.0118090.s003.docx]

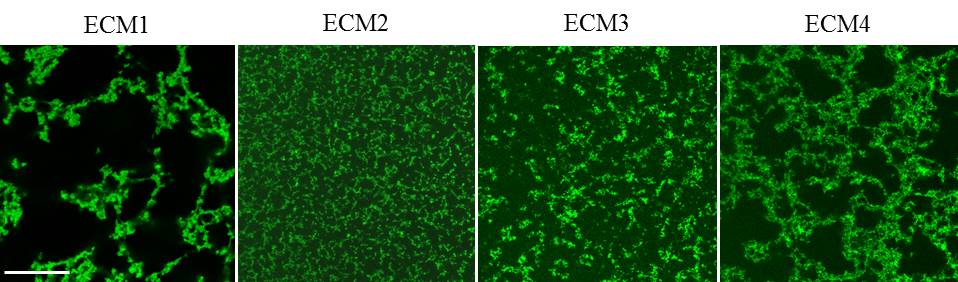


**Figure S3.** Micromorphology of the second batch of the ECM variants as determined by confocal fluorescence microscopy. Representative staining of the matrix component collagen IV. The scale bar in the upper left image denotes 50 µm and applies to all images.
