# Supplementary material for: The Biophysical Properties of Basal Lamina Gels Depend on the Biochemical Composition of the Gel
Source: PLoS One. 2015 Feb 17;10(2):e0118090. doi: 10.1371/journal.pone.0118090 (PMC4331274; doi:10.1371/journal.pone.0118090)
Supplement: S4 Fig — The storage moduli G’ (full circles) dominate over the loss moduli G” (empty circles) for all gel variants. Moreover, the storage moduli of all gels are nearly constant over two decades of frequency. This allows us to define a plateau modulus G 0 for each of the gels: G 0, ECM1 = 3 Pa, G 0, ECM2 = 11 Pa, G 0, ECM3 = 3 Pa, G 0, ECM4 = 4 Pa. The measurement was performed on a stress-controlled macrorheometer (MCR 302, Anton Paar, Graz, Austria) with a 25 mm plate-plate geometry at a plate separation of 200 μm after 30 min gelation time at 37°C. (DOCX) [file pone.0118090.s004.docx]

**
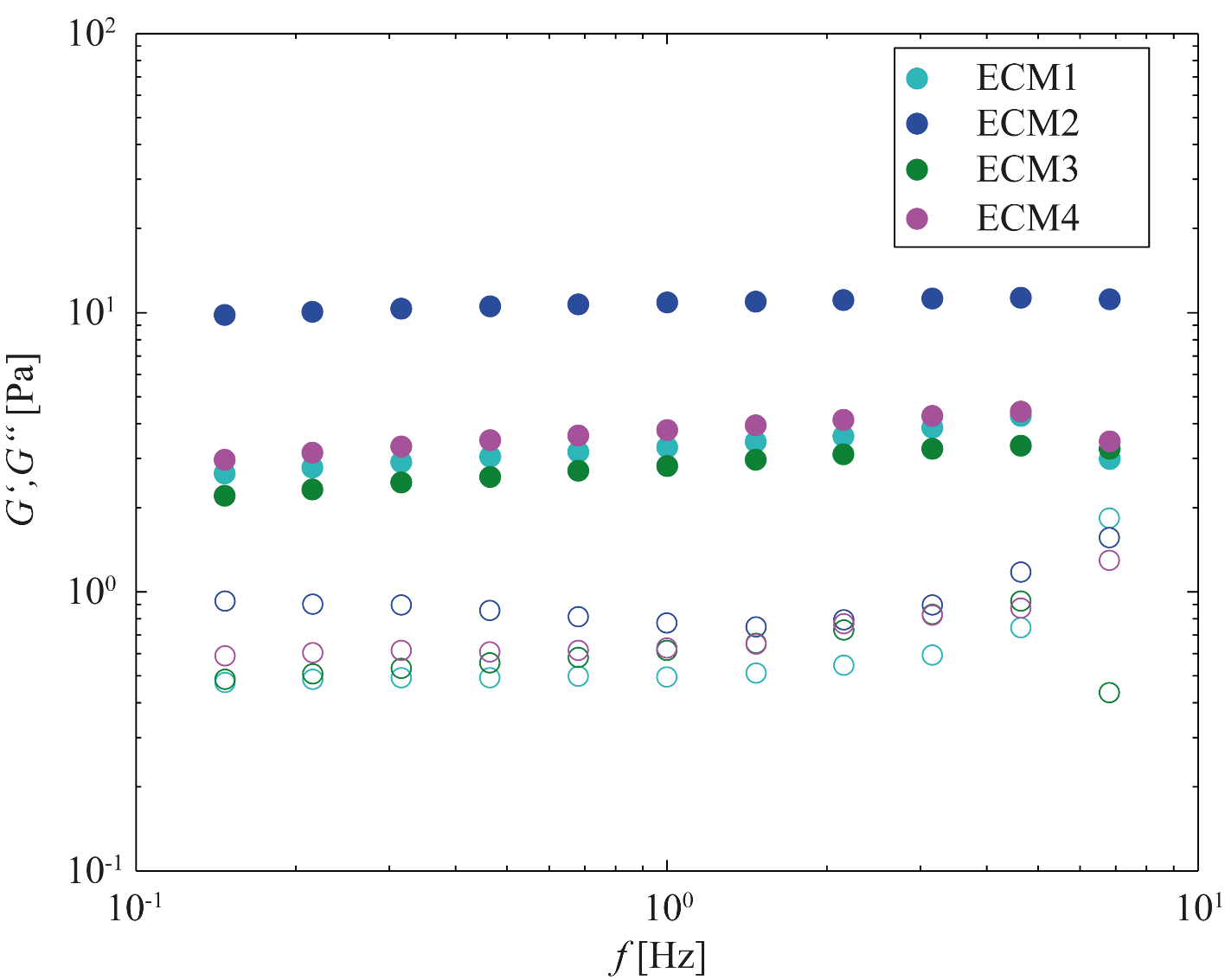
**

**Figure S4.** Viscoelastic frequency spectrum obtained for the basal lamina variants. The storage moduli G’ (full circles) dominate over the loss moduli G’’ (empty circles) for all gel variants. Moreover, the storage moduli of all gels are nearly constant over two decades of frequency. This allows us to define a plateau modulus *G*_0_ for each of the gels: *G*_0,ECM1_ = 3 Pa, *G*_0,ECM2_ = 11 Pa, *G*_0,ECM3_ = 3 Pa, *G*_0,ECM4_ = 4 Pa. The measurement was performed on a stress-controlled macrorheometer (MCR 302, Anton Paar, Graz, Austria) with a 25 mm plate-plate geometry at a plate separation of 200 µm after 30 min gelation time at 37 °C.
