# Supplementary material for: The Biophysical Properties of Basal Lamina Gels Depend on the Biochemical Composition of the Gel
Source: PLoS One. 2015 Feb 17;10(2):e0118090. doi: 10.1371/journal.pone.0118090 (PMC4331274; doi:10.1371/journal.pone.0118090)
Supplement: S5 Fig — Again, ECM2 shows a significant higher G’ than the other three gel variants. The error bar denotes the error of the mean. (DOCX) [file pone.0118090.s005.docx]

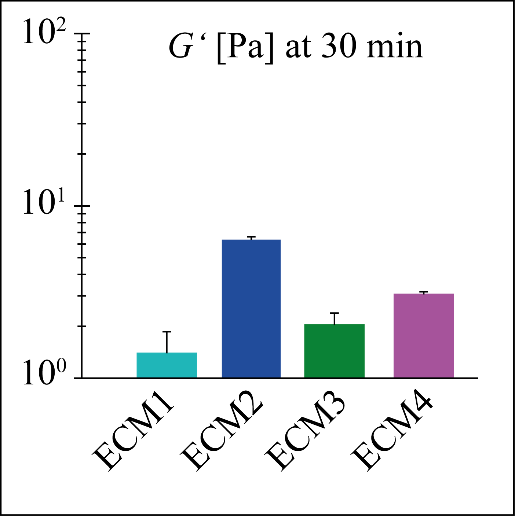


**Figure S5.** Rheological results for the second batch of the gels. Again, ECM2 shows a significant higher *G’* than the other three gel variants. The error bar denotes the error of the mean.
