# Supplementary material for: The Biophysical Properties of Basal Lamina Gels Depend on the Biochemical Composition of the Gel
Source: PLoS One. 2015 Feb 17;10(2):e0118090. doi: 10.1371/journal.pone.0118090 (PMC4331274; doi:10.1371/journal.pone.0118090)
Supplement: S6 Fig — There was no second batch of growth factor reduced ECM1 available therefore we used non-growth factor reduced (ngfr) ECM1 as a control. In ECM1 there is an additional band at 50 kDa which was not detected in the other ECMs. (DOCX) [file pone.0118090.s006.docx]

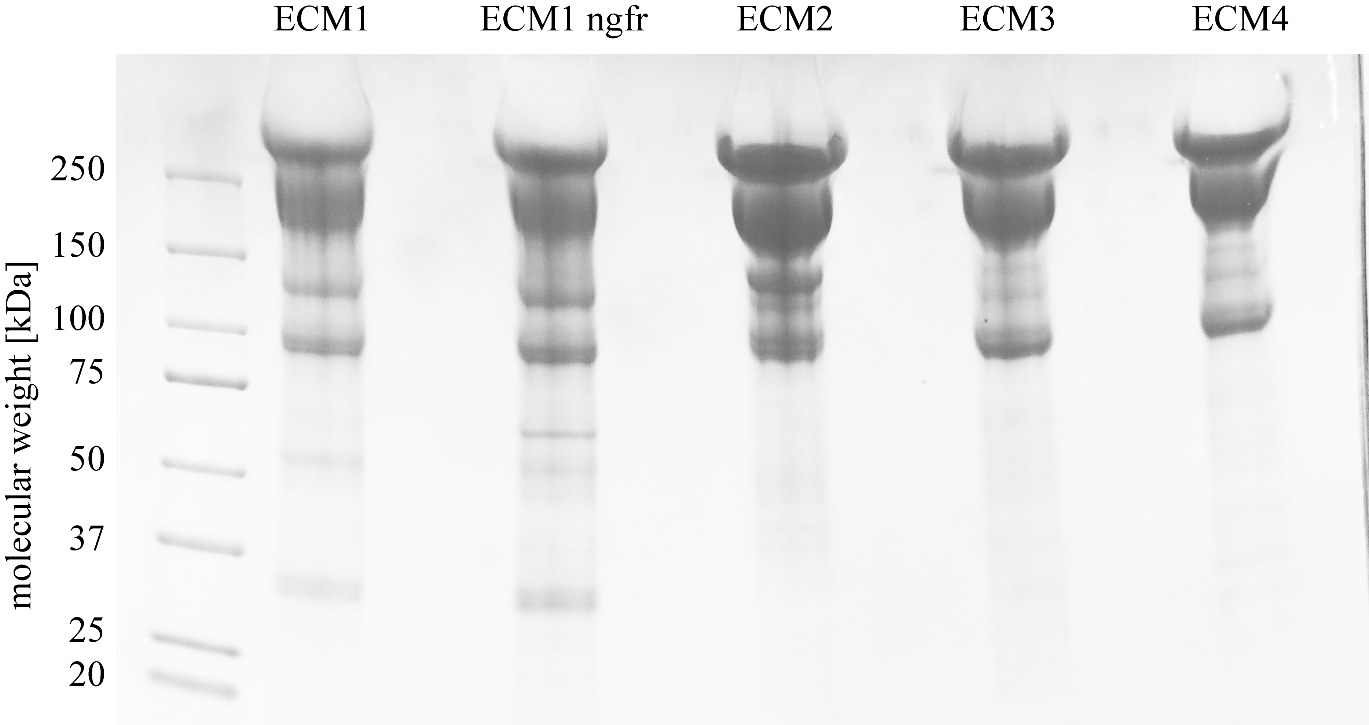


**Figure S6**. SDS-PAGE for second batch. There was no second batch of growth factor reduced ECM1 available therefore we used non-growth factor reduced (ngfr) ECM1 as a control. In ECM1 there is an additional band at 50 kDa which was not detected in the other ECMs.
