# Supplementary material for: The Biophysical Properties of Basal Lamina Gels Depend on the Biochemical Composition of the Gel
Source: PLoS One. 2015 Feb 17;10(2):e0118090. doi: 10.1371/journal.pone.0118090 (PMC4331274; doi:10.1371/journal.pone.0118090)
Supplement: S7 Fig — The entactin blot shows non-specific bands at 130 kDa, 110 kDa and 100 kDa, which were identified by Paulsson et al (Purification and structural characterization of intact and fragmented nidogen obtained from a tumor basement membrane, Eur. J. Biochem. 156, 467–478 (1986)), as entactin fragments obtained under conditions with less stringent control of endogenous proteolysis. The unspecific bands occurring in the laminin blot might show laminin B1 and B2 (>200 kDa) as well as shorter proteolytic laminin fragments (130 and 72 kDa). Short proteolytic fragments might also occur in the collagen IV blot (72 kDa). This blot additionally shows non-specific bands in the region between 300 kDa and 180 kDa, which might be explained by cross-reactivity of the collagen IV-antibody with laminin and fibronectin. The non-specific bands in the fibronectin blot might be also due to short proteolytic fragments. (DOCX) [file pone.0118090.s007.docx]

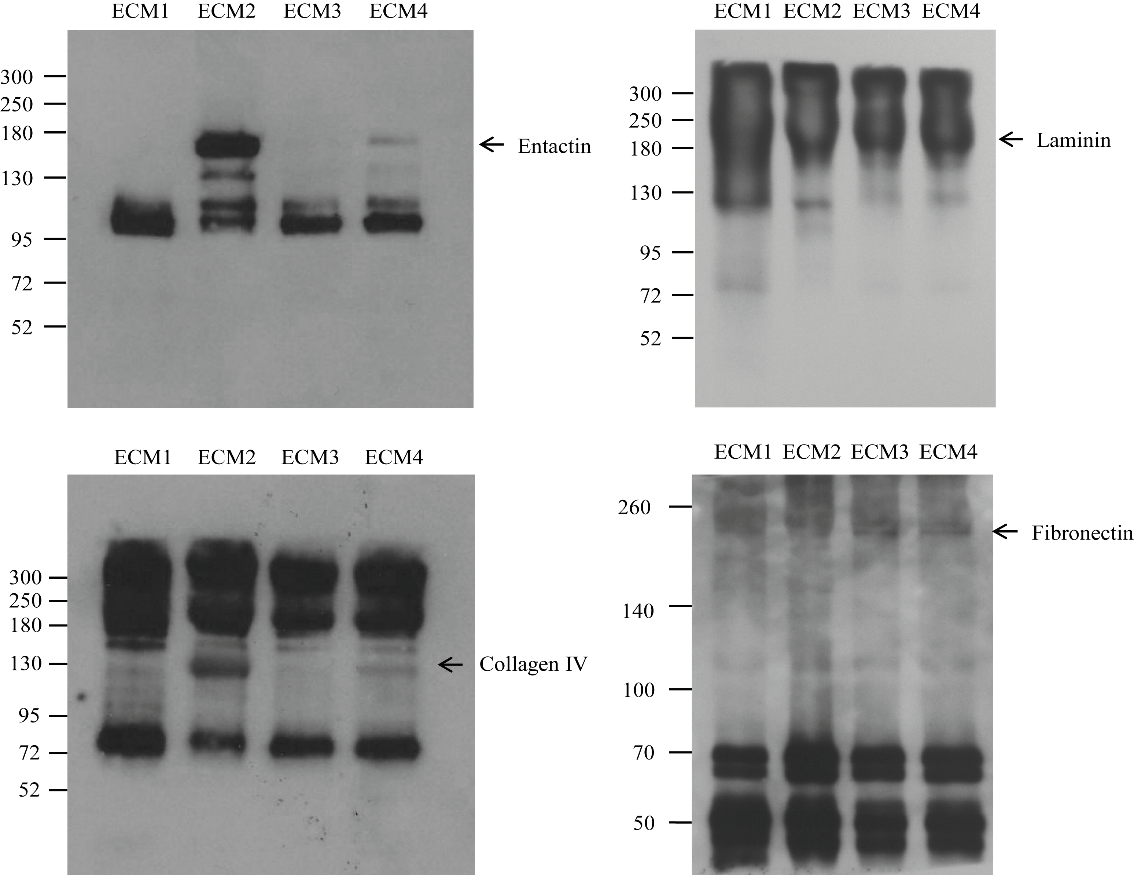


**Figure S7.** Uncropped blots for detection of the ECM proteins entactin, laminin, collagen IV and fibronectin. The entactin blot shows non-specific bands at 130 kDa, 110 kDa and 100 kDa, which were identified by Paulsson et al (*Purification and structural characterization of intact and fragmented nidogen obtained from a tumor basement membrane*, Eur. J. Biochem. 156, 467-478 (1986)), as entactin fragments obtained under conditions with less stringent control of endogenous proteolysis. The unspecific bands occurring in the laminin blot might show laminin B1 and B2 (>200 kDa) as well as shorter proteolytic laminin fragments (130 and 72 kDa). Short proteolytic fragments might also occur in the collagen IV blot (72 kDa). This blot additionally shows non-specific bands in the region between 300 kDa and 180 kDa, which might be explained by cross-reactivity of the collagen IV-antibody with laminin and fibronectin. The non-specific bands in the fibronectin blot might be also due to short proteolytic fragments.
