# Supplementary material for: The Biophysical Properties of Basal Lamina Gels Depend on the Biochemical Composition of the Gel
Source: PLoS One. 2015 Feb 17;10(2):e0118090. doi: 10.1371/journal.pone.0118090 (PMC4331274; doi:10.1371/journal.pone.0118090)
Supplement: S8 Fig — Densiometric analysis of fibronectin, laminin, entactin and collagen IV signals. The error bars denote the standard deviations as obtained from three independent gel runs. ECM2 shows again the significantly highest amount of entactin. (DOCX) [file pone.0118090.s008.docx]

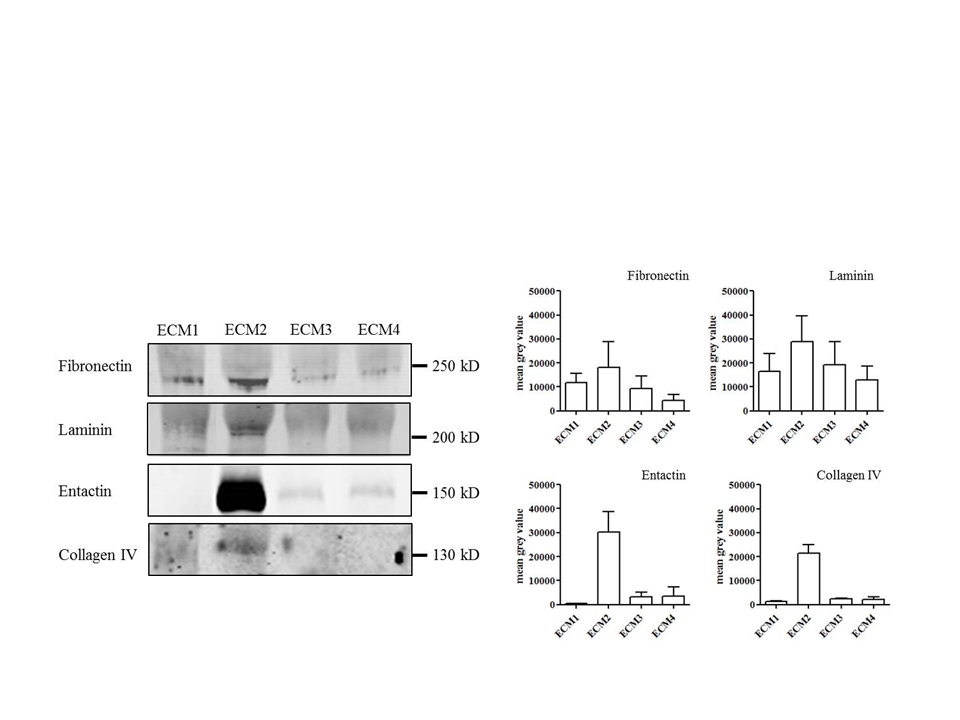


**Figure S8.** The content of fibronectin, laminin, entactin and collagen type IV in the second batch of the four different ECM gels is analyzed by western blot. Densiometric analysis of fibronectin, laminin, entactin and collagen IV signals. The error bars denote the standard deviations as obtained from three independent gel runs. ECM2 shows again the significantly highest amount of entactin.
