# Supplementary material for: The Biophysical Properties of Basal Lamina Gels Depend on the Biochemical Composition of the Gel
Source: PLoS One. 2015 Feb 17;10(2):e0118090. doi: 10.1371/journal.pone.0118090 (PMC4331274; doi:10.1371/journal.pone.0118090)
Supplement: S1 Table — Again, amine-terminated as well as carboxyl-terminated particles are immobile in all ECMs. ECM1, ECM3 and ECM4 show a similar fraction of diffusing PEGylated particles whereas in ECM2 only immobile particles are detected. (DOCX) [file pone.0118090.s009.docx]

**Table S1.** Analysis of particle tracking experiments for a second batch of the gels. Again, amine-terminated as well as carboxyl-terminated particles are immobile in all ECMs. ECM1, ECM3 and ECM4 show a similar fraction of diffusing PEGylated particles whereas in ECM2 only immobile particles are detected.

|  | Amine | Carboxyl | PEG |
| --- | --- | --- | --- |
| ECM1 | 0 | 0 | (84 ± 12) % |
| ECM2 | 0 | 0 | 0 % |
| ECM3 | 0 | 0 | (71 ± 7) % |
| ECM4 | 0 | 0 | (74 ± 2) % |
