# Supplementary material for: The Biophysical Properties of Basal Lamina Gels Depend on the Biochemical Composition of the Gel
Source: PLoS One. 2015 Feb 17;10(2):e0118090. doi: 10.1371/journal.pone.0118090 (PMC4331274; doi:10.1371/journal.pone.0118090)
Supplement: S2 Table — Database: NCBInr 20140323, Taxonomy: Mus musculus, Type of search: Peptide mass fingerprint, Enzyme: Trypsin (DOCX) [file pone.0118090.s010.docx]

**Table S2.** Results from mass spectroscopy analysis of the additional band of ECM1. Database: NCBInr 20140323, Taxonomy: Mus musculus, Type of search: Peptide mass fingerprint, Enzyme: Trypsin

|  | **Mass** | **Score** | **Expected** | **Matches** |
| --- | --- | --- | --- | --- |
| gi\|52858 | 37712 | 63 | 0.087 | 8 |
| Hartl,L., Oberbaumer,I. and Deutzmann,R., *The N terminus of laminin A chain is homologous to the B chains*, Eur. J. Biochem. 173 (3), 629-635 (1988) | | | | |
| gi\|74224878 | 37746 | 46 | 4.1 | 8 |
| gi\|400977322 | 58595 | 31 | 1.5e+02 | 8 |
| gi\|111305466 | 63060 | 27 | 3.7e+02 | 7 |
| gi\|119226206 | 11651 | 45 | 5.2 | 5 |
| gi\|6531381 | 10720 | 32 | 1.2e+02 | 4 |
| gi\|74152705 | 12396 | 43 | 9.1 | 4 |
| gi\|74224092 | 15468 | 36 | 39 | 4 |
| gi\|148666045 | 54791 | 34 | 63 | 6 |
| gi\|15126700 | 33604 | 33 | 87 | 6 |
| gi\|19353516 | 48696 | 26 | 4.7e+02 | 6 |
| gi\|26346418 | 48757 | 26 | 4.7e+02 | 6 |
| gi\|269308251 | 48742 | 26 | 4.7e+02 | 6 |
| gi\|148694651 | 3714 | 33 | 95 | 2 |
| gi\|9837303 | 12897 | 32 | 1.2e+02 | 3 |
| gi\|347943574 | 6034 | 31 | 1.3e+02 | 3 |
| gi\|12853295 | 22326 | 30 | 1.7e+02 | 4 |
| gi\|18845005 | 17541 | 30 | 1.9e+02 | 4 |
| gi\|159162515 | 14644 | 30 | 1.9e+02 | 3 |
| gi\|148685215 | 33776 | 29 | 2e+02 | 4 |
| gi\|83627687 | 11276 | 29 | 2.2e+02 | 3 |
| gi\|289526642 | 4440 | 29 | 2.3e+02 | 2 |
| gi\|568912134 | 54738 | 29 | 2.3e+02 | 5 |
| gi\|568912132 | 55153 | 28 | 2.4e+02 | 5 |
| gi\|568912128 | 55368 | 28 | 2.5e+02 | 5 |
| gi\|568912108 | 60167 | 27 | 3.5e+02 | 5 |
| gi\|568912106 | 60582 | 27 | 3.7e+02 | 5 |
| gi\|568912104 | 60796 | 27 | 3.8e+02 | 5 |
| gi\|568912102 | 61195 | 26 | 3.9e+02 | 5 |
| gi\|568935374 | 102356 | 28 | 2.4e+02 | 6 |
| gi\|26343501 | 108941 | 27 | 3.1e+02 | 6 |
| gi\|164519057 | 108914 | 27 | 3.1e+02 | 6 |
| gi\|2558835 | 108928 | 27 | 3.1e+02 | 6 |
| gi\|50511047 | 64568 | 28 | 2.5e+02 | 6 |
| gi\|18044474 | 55932 | 28 | 3e+02 | 5 |
| gi\|67010061 | 56108 | 27 | 3.2e+02 | 5 |
| gi341940401 | 55727 | 27 | 3.6e+02 | 5 |
| gi\|4249595 | 55743 | 27 | 3.6e+02 | 5 |
| gi\|254281186 | 59054 | 26 | 4.3e+02 | 5 |
